# Scrap the Food Waste: An Investigation of the Effect of Sociodemographic Factors and Digital Activism on Food Waste Prevention Behavior

**DOI:** 10.3390/foods15030456

**Published:** 2026-01-28

**Authors:** Maria Piochi, Riccardo Migliavada, Maria Giovanna Onorati, Franco Fassio, Luisa Torri

**Affiliations:** University of Gastronomic Sciences, 12042 Pollenzo, Italy; m.piochi@unisg.it (M.P.); m.onorati@unisg.it (M.G.O.); f.fassio@unisg.it (F.F.); l.torri@unisg.it (L.T.)

**Keywords:** consumer behavior, food waste knowledge, food waste attitude, information sensitivity, behavioral change, social media activism

## Abstract

Food waste is a persistent global concern, requiring behavioral and systemic responses from consumers. The current study investigated the effect of sociodemographic factors and digital activism on food waste prevention behavior. Data from 390 respondents living in Italy (65% females, from 18 to 75 years old, grouped into four generations) were collected through an online survey covering these sections: sociodemographic variables, digital activism, knowledge, attitudes, and food waste behaviors. A Food Waste Prevention Index (FWPI) was computed to assess self-reported adherence to waste-reducing practices, and differences across three groups identified through tertiles were tested. Women displayed higher levels of digital activism; Gen Z was the most engaged generation in seeking information about food, while interest in food issues declined with age. Gender, geographical area, and dietary orientation significantly influenced food waste prevention, with women, rural residents, and individuals adopting flexitarian or vegetarian diets tending towards more virtuous behavior (higher FWPI). According to digital activism, less virtuous waste behavior (lower FWPI) was associated with a lower social media and apps usage frequency. Furthermore, higher FWPI individuals self-reported stronger sensitivity to sustainability-related topics such as circular economy, short food chains, and ethical or environmental motivations for vegetarianism. Overall, awareness and digital activism may synergistically foster more responsible food consumption, and targeted communication and digital tools can effectively support household food waste reduction strategies.

## 1. Introduction

Since the FAO reported that approximately 30% of food produced is wasted [1], Food Loss and Waste (FLW) has become a central issue linking the challenges of feeding a growing population, sustaining supply chain incomes, and meeting climate commitments [2,3,4]. This phenomenon represents a profound ethical and structural inefficiency [5], particularly given the paradoxical coexistence of wastage with widespread global food insecurity [6]. Furthermore, FLW is exacerbated by economic and climatic shocks [7] and remains a primary driver of environmental degradation, contributing to 8–10% of greenhouse gas emissions and resulting in significant land [8] and water loss [9].

FAO estimates [1] laid the groundwork for the United Nations Sustainable Development Goal (SDG) 12, which targets halving global per capita food waste at retail and consumption level by 2030, while also reducing losses along production and supply chains [10]. According to definitions provided by international bodies [8], food waste refers to food originally intended for human consumption that is discarded or allowed to deteriorate along the supply chain due to human behavior. At the domestic level, this description encompasses the avoidable waste that is “edible” (e.g., perfectly edible food discarded for cultural, esthetic, or habitual reasons, such as peels, leaves, stems, rinds, etc.) and inevitable waste that is “inedible” (e.g., inedible or unpredictably spoiled parts). Such categories of waste outlined here allow for a wide margin of interpretation depending on technological and economic developments, culture, and habits, typical of a country [11]. In 2022, approximately 1.05 billion tons of food were wasted globally, with the household sector accounting for 59% of the total [9]. Within the Italian context, this corresponds to 98 kg of food waste per capita annually [12].

A 2023 OECD (Organization for Economic Co-operation and Development) survey across nine countries identified spoilage and expiration dates as primary drivers of waste [13]. This suggests that household food waste is predominantly characterized by “non-action”—such as failure to cook or plan efficiently—rather than active intervention [14]. While often linked to behavioral lapses like poor stock organization, waste is also shaped by sociodemographic variables, product attributes, and local contexts [15]. Consequently, food waste must be understood not merely as material loss, but as a structural indicator of broader systemic imbalances [16], deeply rooted in established daily routines.

The topic of food waste among consumers has been increasingly tackled in academic studies, highlighting its complexity. Indeed, it is a multi-faceted phenomenon that cannot be attributed to single variables [17]. Much research on consumer’s perspective has focused on developing models explaining food waste among adults, considering the theories of value, belief, and norm and the theory of planned behavior [18], or considering aspects related to food product specificity (label information, package size, product category, etc.) [19]. Moreover, an extended literature recently explored the topic of the food waste in school canteens, focusing on children as the target audience [20,21,22,23,24,25]. Among interesting results found in schools, integrated approaches are recommended to minimize food waste that involve all stakeholders including municipality, schools, parents and families (including also needy families), non-profit organizations, local health authorities, and catering companies [22]. Indeed, the domestic dimension of food waste is among the most relevant. The topic of food waste is a relevant issue at the domestic level, and it has recently been reviewed [17], showing that food waste can originate and may rely on a non-optimal management of one or more phases including planning, shopping, storing, cooking, eating, and leftover management [17]. In a study conducted in Italy, sociality (cohabitation with a partner or living in a large size family) and the attendance of certain academic disciplines (specifically, scientific and technological fields) were positively associated with adopting anti-waste behaviors at the domestic level, highlighting the role of community and educational backgrounds in shaping attitudes towards food waste [26].

Currently, several methods are envisaged to estimate the level of food wastage in households, including surveys, interviews, and diaries [27,28]. A comprehensive, integrated, and systemic approach, capable of combining education, effective policies, and community initiatives adapted to specific demographic and socio-economic contexts, is a prerequisite for achieving a significant reduction in food waste at the household level [29].

On the other hand, digital applications and tools have increasingly become a part of our daily lives and offer a promising strategy to shift food systems towards more sustainable systems mainly through different approaches that include information provisioning (e.g., carbon footprint learning, serious games), consumption monitoring (e.g., food waste reduction, dietary tracking), and mindset transformation [30]. Recently, the promotion of sustainable eating behavior change, particularly reducing food waste, through digital interventions has been reviewed [31], with a general finding that social media may have a positive impact by raising awareness or contributing to consumer food waste reduction. A recent review considered food waste in the sector of tourism and hospitality and identified three types of interventions that can be addressed by social media, respectively: (1) antecedent (social norm, social support, and awareness), (2) moderator (platform nudge, sharing, and identity), and (3) consequence to the tourist experience (gamification and feedback) [32]. A study conducted with Malaysian customers of retail fast-food chains found that social media food waste advertising positively affects customers’ intentions not to waste food directly and indirectly through two mediators: awareness of food waste consequences and customer–brand communal engagement [33].

However, studies approaching the topic in a multi-disciplinary way and merging the perspective of consumer science with digital interventions in reducing food waste are still lacking if considering the domestic level. The current study tries to fill this gap by applying a multi-disciplinary approach that considers the consumer perspective (sensory and consumer science) and by integrating it with a sociological perspective that focuses on the impact to the adoption of social media with a circular vision.

Based on the aforementioned aspects, the general aim of the present article was to provide a comprehensive understanding of the factors shaping household food waste prevention through an analysis of daily food management practices. In particular, the specific aims were (1) to investigate daily behaviors and practices in food management and waste prevention and (2) to explore the influence of sociodemographic characteristics, digital activism, knowledge, individual sensitivity, and attitude towards the issue of food waste on behaviors to prevent and reduce household waste. The analysis’s findings offer empirical evidence that can inform the design of communication campaigns and prevention policies aimed at overcoming the identified obstacle.

## 2. Materials and Methods

### 2.1. Online Survey

An online self-reported survey composed of 52 questions was designed in the Italian language using the software Qualtrics^®^ (Qualtrics, Provo, UT, USA). Data were collected anonymously between December 2024 and February 2025. The online survey consisted of five separate sections with the following order: (i) sociodemographic characteristics (12); (ii) Digital Activism (12); (iii) Food Waste Knowledge (5); (iv) Sensitivity and Attitude (5); (v) Food Waste Behaviors and Practices (18). Prior to data collection, the questionnaire underwent an internal pre-testing phase by the research team to ensure technical functionality and verify the estimated completion time provided by the Qualtrics platform (15 min). No external pilot study was conducted for item clarity, as the survey items were derived from previously validated scales found in the literature. The complete questionnaire is reported in Supplementary Materials (S1).

#### 2.1.1. Sociodemographic Questions

All respondents were asked to provide their *gender* (female; male; non-binary; I prefer not to say), *age*, *nationality*, if they are *living in Italy* (Yes, permanently; Yes, but I spend long periods (whole months) in another country; No, I live permanently in another country), in which *Italian region* they live, the context of *residence* (village/rural context (<10,000 inhabitants); town (10,000–70.000 inhabitants); city (>70,000 inhabitants)), the *level of education* (middle school or below; high school diploma; bachelor’s or postgraduate degree; master’s or doctoral degree), *household* members (parents or other members of the family of origin; children; wife/husband or cohabiting partner; wife/husband or cohabiting partner and children; friends/roommates; students or fellow roommates; none, I live alone), their *occupational status* (employed full-time, part-time, temporary, casual, self-employed; student; not employed/unemployed), their *economic status* (I am completely self-sufficient, I support myself; I am partially self-sufficient, my family (relatives or partners) supports me; I am not self-sufficient, my family (relatives or partners) supports me completely; I am not self-sufficient (I take welfare), and their *diet* (omnivorous; flexitarian; vegetarian; vegan).

#### 2.1.2. Digital Activism and Food-Related Digital Engagement

The aim of this section is to explore and validate digital activism in the sample as a constellation of diverse yet interrelated observable digital practices, rather than as a homogeneous attitudinal construct, in line with a practice-based approach to media [34]. Accordingly, the items were designed to capture what users do with digital platforms, which platforms they use, how frequently they engage with them—thus incorporating time as routinization, as theorized in practice theory [35]—and the purposes for their engagement (e.g., checking, scrolling, posting, or seeking information related to dietary patterns or food shopping). These indicators therefore function as descriptive and analytical markers of practice, rather than as measures of personal values or motivations. Accordingly, questions related to digital activism examined the frequency of social media use (Instagram, Facebook, X (ex-Twitter), Thread, LinkedIn, TikTok); the average frequency of checking one’s preferred social media profile (1 = more than once per hour; 6 = once per week); the frequency of certain activities on social media regarding posts and contents self-created or created by others (viewing/reading, creating, sharing); frequency of posting content on one’s preferred social media platform; frequency of using social media to obtain food-related information; frequency of using social media to obtain food waste-related information; number of contacts/followers on one’s preferred social media platform (1 = 0–500; 7 = more than 100,000); number of followed accounts on the preferred social media platform (1 = 0–500; 6 = 50,000 or more); frequency of use of daily planner apps, respectively, for food consumption and for grocery shopping; frequency of use of apps for food delivery; and frequency of use of apps like TooGoodToGo, Phenix, MyFood, and Wastemeter. Where not specified, the above frequencies were collected on a five-point scale (1 = Never; 5 = Always).

#### 2.1.3. Food Waste Knowledge Assessment

Respondents’ knowledge of food waste was measured using five multiple-choice questions addressing widely recognized aspects of the issue and selected by experts. The questions were derived from the UNEP Food Waste Index Report (2024) and the FAO report, The State of Food and Agriculture (2019). Each question presented four response options, of which only one was correct. The items addressed five specific dimensions: (1) the estimated percentage of global food production wasted annually; (2) the sector contributing most to global food waste; (3) the average annual per capita food waste in high-income countries; (4) the most effective behaviors for reducing household waste; and (5) the primary drivers of waste in the retail sector in high-income countries. Correct responses were awarded one point, while incorrect answers received zero. The sum produced an aggregate knowledge score (range: 0–5), where higher values reflect greater awareness and understanding of food waste.

#### 2.1.4. Sensitivity and Attitude

To assess sensitivity and attitudes, respondents completed five sets of questions using a 5-point scale (1 = Not at all important; 5 = Very important). Firstly, they rated how important it was to be informed about the following topics: food waste, vegetarianism, circular economy, product seasonality, and origin. Secondly, they evaluated the perceived importance of different actors in reducing food waste: food distributors (e.g., stores, large-scale retailers, importers), agricultural production, the food processing industry, the third sector (e.g., NGOs, social enterprises), the public sector (e.g., cities, states), and themselves [36]. Thirdly, they rated the importance of specific food characteristics, including whether a product is local/regional, seasonal, from nearby (local), organic, of recognized quality, fair trade, and whether it reflects environmental, economic, and social sustainability [37]. Fourthly, they assessed the importance of potential reasons for following a vegetarian diet, namely, personal health, desire to discover new flavors, need to lose weight, environment/climate, animal welfare, taste, curiosity, influence of cohabiting vegetarians, economic considerations, concern for the Third World, spiritual/religious beliefs, social pressure, viewing meat consumption as a transitional phase, and no reason [38]. Finally, willingness to reconsider one’s consumption habits to reduce food waste was measured on a five-point frequency scale (1 = Never; 5 = Always). Higher scores indicate greater perceived importance or willingness.

#### 2.1.5. Food Waste Behaviors and Practices

This section of the survey was composed of two parts. The first part consisted of two questions investigating digital engagement in relation with behaviors and practices related to food waste, while the second aimed to measure the tendency toward food waste production. The digital engagement on four topics—food waste, vegetarianism, circular economy and kitchen recovery practices, and product seasonality and origin—was measured with two items: frequency of online sharing (“*How often do you share information, opinions, or viewpoints* via *digital devices and social networks about the following topics?*”) and frequency of online information seeking on digital devices and social media (“*Regarding the following topics, how often do you read news, in-depth coverage,* etc., *on digital devices and social media?*”). Responses were provided on a 5-point scale (1 = Never; 5 = Always).

To measure behaviors and practices related to food waste, we developed a 16-item questionnaire on a 5-point Likert scale (1 = Never, 5 = Always). Six questions (1–6) focused on food waste reducing practices (e.g., *Do you plan your meals before shopping?*) [15,17,39,40,41,42,43,44], three (7–9) on alimentary behaviors (e.g., *Do you prepare different dishes every day?*) [43,45], two (10–11) on circular economy practices (e.g., *Do you throw away leftovers because no one wants to eat them?*) [39,42], and five (12–16) on food waste increasing practices (e.g., *Do you forget about food in the fridge or freezer until it is too old to eat?)* [17,39,41,42,44,45,46]. Eight questions (9–16) were reverse scored. Higher total scores reflected greater adherence to practices and behaviors that reduce food waste, while lower scores indicated a tendency toward food waste production.

### 2.2. Participants

Participants were recruited from the “Scrap The Food Waste” community of AWorld, a digital platform designed to drive positive impact through the use of gamification [47]. A total of 534 responses were collected, of which 132 were excluded because they were incomplete (91) or because participants did not give their consent to participate (40). Moreover, 12 responses were discarded because they did not meet the inclusion criteria (i.e., age between 18 and 75). In particular, eight were not considered because the subjects were under the age of 18 and therefore ineligible to sign informed consent and respond to the survey; four were discarded because they were older than 75 years and outside our inclusion criteria (18–75), as this category of people is more likely to have particular age-related food habits and is less suitable for online surveys conducted on technological devices. The final sample consisted of 390 respondents (mean age 39.5, SD 12.5; 65.6% females). Table 1 provides a detailed description of the sociodemographic data of respondents. The population was composed of a majority of females, mostly Italians permanently living in Italy. Various generations joined the survey, with the Millennials (30–45 years old) being the most represented. Most participants were employed and with a high educational level. Concerning geographical provenance, most were from the North-West, with almost half of the population living in cities. Regarding food habits, respondents were quite balanced between omnivores and flexitarians, with the minority adopting vegetable-oriented diets. The sample showed predominantly low to medium usage of digital tools related to food and especially for food waste information (46%). Daily apps for food delivery, grocery, and food waste management were marginally used (≥70% fall in the low category). Apart from the variable frequency of social media use to get informed, only a small percentage reported high activism across all digital behaviors (generally ≤ 10%), indicating limited adoption of food-related digital solutions among the population.

The research was approved by the Ethics Committee of the University of Gastronomic Sciences (Ethics Committee proceedings n. UNISG18122024 of 18 December 2024), and it was carried out in accordance with the international ethical guidelines for research involving humans established in the Declaration of Helsinki. All subjects provided written informed consent before beginning the survey.

### 2.3. Data Analysis

#### 2.3.1. Data Pre-Processing

The variable *gender* was consolidated into three categories: female, male, and non-binary/other (including “non-binary” and “I prefer not to say”). Respondents were grouped into four generational cohorts according to their age: (i) Generation Z (≤29 years old, y.o.); (ii) Millennials (30–45 y.o.); (iii) Generation X (46–61 y.o.); and (iv) Baby Boomers (62–78 y.o.).

*Level of education* was classified into three groups: low (middle school diploma or less), medium (high school diploma), and high (bachelor’s degree or higher). The *Italian regions* of residence were aggregated into four macro-areas according to NUTS areas (EU and of the EC No 1059/2003): North-West (Liguria, Lombardia, Piemonte, Valle d’Aosta); North-East (Emilia Romagna, Friuli Venezia Giulia, Trentino-Alto Adige, Veneto); Center (Lazio, Marche, Umbria, Toscana); and South and Islands (Abruzzo, Basilicata, Calabria, Campania, Molise, Puglia, Sardegna, Sicilia). Dietary profiles were aggregated into three categories based on frequency and amount of meat consumption: does not eat meat (vegetarian, vegan), eats little meat (flexitarian, pescatarian), and eats meat (omnivorous).

Responses related to digital activism (1 = Never; 5 = Always) (Table 1) were re-coded into three levels: Low (Never, Rarely), Medium (Sometimes), and High (Often, Always). The re-coding procedure was applied to the following items: “*How often do you use social media to inform yourself about food waste?*”, “*How often do you use apps that provide a daily planner of how much food to eat?*”, “*How often do you use apps that provide a daily planner for grocery shopping?*”, “*How often do you use food delivery apps?*”, and “*How often do you use apps such as TooGoodToGo, Phenix, MyFood, Wastemeter?*”.

Finally, distinct profiles of digital activism were identified in the population based on the frequency (1 = Never; 5 = Always) of use of the most popular social media platforms (*Frequency of use of Instagram, Facebook, X (formerly Twitter), Threads, LinkedIn, TikTok*), with particular attention given to the level of engagement in digital practices related to food and food waste reduction (*Frequency of use of social media to get informed about food; frequency of use of “daily meal planner” apps; frequency of use of “daily grocery shopping planner” apps; frequency of use of food delivery apps; frequency of use of anti-waste apps such as TooGoodToGo,* etc.). In particular, respondents who “never” or “rarely” used the social media platforms (i.e., Instagram, Facebook, X, Threads, LinkedIn, TikTok, daily meal planners, grocery shopping planners, and anti-waste apps) were classified within “low DA” digital activism profiles; those who used them “sometimes” within “medium DA” profiles; and those who used them “often” or “always” within “high DA” profiles.

#### 2.3.2. Statistical Analysis and Food Waste Prevention Index Computation

The statistical software used for all analyses were R (2024.09.1), Xlstat Sensory 2025.1.3 (Addinsoft, New York, NY, USA), and SPSS (V. 29.0.1.0). Different ANOVA models were applied to assess how the main sociodemographic factors (i.e., gender, age, education, economic status, and geographic area) affected different aspects of digital activism (including the use of different digital platforms and apps and different activities related to digital profiling—such as digital contents consumption) by adopting the sociodemographic factor as a fixed factor.

To assess the unidimensionality of the questionnaire Food Waste Behaviors and Practices (16 items rated on a 5-point Likert scale as reported in Section 2.1.5; 1 = Never, 5 = Always), we conducted an Exploratory Factor Analysis (EFA). Prior to analysis, items were oriented so that higher scores indicated greater adherence to behaviors that reduce food waste (reverse-coded where necessary). Results indicated that only items 10–16 loaded coherently on a single factor, whereas items 1–9 showed heterogeneous loadings and/or cross-loadings inconsistent with a unique domain. Accordingly, for subsequent analyses we computed a composite index (Food Waste Prevention Index—FWPI) as the sum of responses to items 10–16 (Cronbach’s alpha = 0.78), with higher scores reflecting stronger adherence to food waste-reducing behaviors and practices.

A multiple linear regression analysis was performed to identify the determinants of the FWPI. The FWPI served as the dependent variable, while the independent variables included respondents’ food waste knowledge score, gender, study level, economic status, dietary profile, household composition, generation, and geographical macro-area.

To compare FWPI across sociodemographic groups, we used the nonparametric Kruskal–Wallis test; when the result of the test was significant (*p* < 0.05), we conducted a post hoc analysis using Dunn’s pairwise rank-based comparisons with Bonferroni-adjusted *p*-values.

Finally, respondents were segmented into three groups (low_FWPI, medium_FWPI, and high_FWPI) using tertile values of the FWPI, indicating different attitudes towards food waste prevention. Chi-square tests were run for all variables to assess differences across groups obtained according to different FWPIs. For variables indicating differences across groups, significance by-cell Fisher’s exact tests were run. Two-way ANOVA models were separately applied to assess the effect of group belonging (low_FW, medium_FW, high_FW = fixed factor; respondent = random factor) to variables concerning the attitude of the groups for food waste behavior and sensitivity to information. ANOVA models conducted on the groups with different FWPIs were followed by Tukey’s test for multiple comparisons of means (*p* < 0.05).

## 3. Results

### 3.1. Sociodemographic Patterns in Digital Activism and Food-Related Digital Engagement

Data presented in Table 2 show that digital activism (DA) varies considerably across platforms, with Instagram displaying the highest levels of engagement, Facebook showing a more mixed distribution, and platforms such as X, Threads, TikTok, and LinkedIn characterized by predominantly low profiles. Passive activities—such as watching, reading, scrolling, and checking one’s profile—were the most widespread, whereas productive activities (creating, commenting, reposting, posting) show lower overall participation. Regarding food-related activism, seeking information about food is relatively balanced across low, medium, and high profiles, while food waste information tends to cluster in low and medium profiles. The use of food-related apps—meal planners, grocery planners, delivery services, and anti-waste apps like TooGoodToGo—shows very low engagement, suggesting limited integration of these digital tools into everyday food practices.

The ANOVA revealed significant differences across groups (*p* < 0.05) and relevant impact of sociodemographic characteristics on user profiles (effect size, η^2^) (Table 3). Moreover, the sociodemographic characteristics significantly influenced digital activism, particularly in relation to food-related digital practices and food waste (Table 3). The strongest effects were observed for gender, generation, and socio-economic status, followed—though to a lesser extent—by educational level and geographical area. Although the effect of gender was generally small to moderate, women displayed higher levels of digital activity than men and non-binary individuals across nearly all areas investigated. Women reported greater use of Instagram (η^2^ = 0.016, *p* = 0.046), confirming their higher familiarity with and participation in visually oriented platforms. The most pronounced gender difference concerns content management (η^2^ = 0.052, *p* < 0.001), with women engaging more frequently in scrolling, reading, and watching, as well as in posting and content sharing (η^2^ = 0.032, *p* = 0.002), indicating a stronger participatory attitude. The strongest gender effect was found for seeking information about food (η^2^ = 0.072, *p* < 0.001), and women showed a higher tendency to look for information on food waste (*p* = 0.001) and to use apps to reduce it (*p* = 0.012; η^2^ = 0.023). These results aligned with prior studies highlighting women’s greater engagement in wellbeing, nutrition, care, and ecological awareness.

Generational differences significantly influenced digital activism, with marked effects at the extremes of the age spectrum. Generation Z (17–29 y.o.) showed the highest levels of activity and diversity of interaction, whereas older generations adopted more selective and traditional behaviors. Instagram was the most popular platform among younger people (η^2^ = 0.148, *p* = 0.001), while Facebook showed the opposite trend (η^2^ = 0.049, *p* = 0.001), being more widely used by Gen X and Millennials. TikTok was the most generation-defined platform (η^2^ = 0.088, *p* = 0.001), used far more by Gen Z, with usage declining sharply with age. The frequency of checking social media profiles also decreased with age (η^2^ = 0.105, *p* = 0.001). Gen Z was the most active, followed by Millennials, while Gen X and Baby Boomers were less engaged. Passive content consumption was widespread but most pronounced among younger users (η^2^ = 0.112), who also shared more content, though this effect was smaller (η^2^ = 0.032). Interest in food-related issues declined significantly with age (*p* = 0.001). Gen Z remained the most engaged in seeking information about food, although no generational differences were found in the use of social media to combat food waste.

Education had a significant effect only on LinkedIn use (*p* = 0.000; η^2^ = 0.099). Individuals with higher education use this platform more frequently than those with lower or medium levels. No other significant associations were found between education and social media use, including those related to food and food waste.

The macro-geographical area exerted a considerable, though not dominant, influence on social media use for activism and participation. Significant regional differences emerged for Facebook, X (formerly Twitter), and LinkedIn, with the latter showing the strongest effect. Facebook usage was slightly higher in the Center and Islands (η^2^ = 0.026, *p* = 0.040), while X showed moderately higher usage in the South (η^2^ = 0.028, *p* = 0.030). For LinkedIn, the geographical effect was highly significant (η^2^ = 0.041, *p* = 0.003). The North-West recorded the highest average use, while the South showed the lowest. This pattern likely reflected structural differences in labor markets, professional opportunities, and networking-oriented digital cultures more established in the North. No significant regional differences were observed in social media uses related to food or food waste.

Economic conditions significantly affected several forms of digital activism, with small but relevant differences (η^2^ between 0.022 and 0.047) across various domains of social media and food management app use (Table 3). On social media, Instagram was more frequently used by individuals who were partially self-sufficient or financially dependent on their families (η^2^ = 0.030, *p* = 0.009), indicating stronger engagement among younger, economically dependent users. LinkedIn followed the opposite pattern, being used more by fully self-sufficient individuals (η^2^ = 0.027, *p* = 0.016), reflecting the link between economic stability and professional networking. TikTok showed the most significant differences (η^2^ = 0.047, *p* < 0.001): usage rose as self-sufficiency decreased, except among benefit recipients, who displayed the lowest engagement. Overall, use was polarized between low activity among the most self-sufficient and high activity among those most dependent on family support.

The frequency of checking social media profiles was higher among those who were not fully self-sufficient but declined among benefit recipients, suggesting that economic status interacts with age. Regarding food management apps, meal-planning apps were used significantly more by economic benefit recipients (*p* = 0.008), indicating greater attention to meal organization, with usage increasing as self-sufficiency declines. Grocery-planning apps also showed significant variation (*p* = 0.004), with the highest use one again among benefit recipients, likely for financial reasons, while similarly high use among self-sufficient individuals may reflect greater awareness of food management and waste prevention.

### 3.2. Influence of Sociodemographic Characteristics on Food Waste Prevention Index

**Figure 1:**
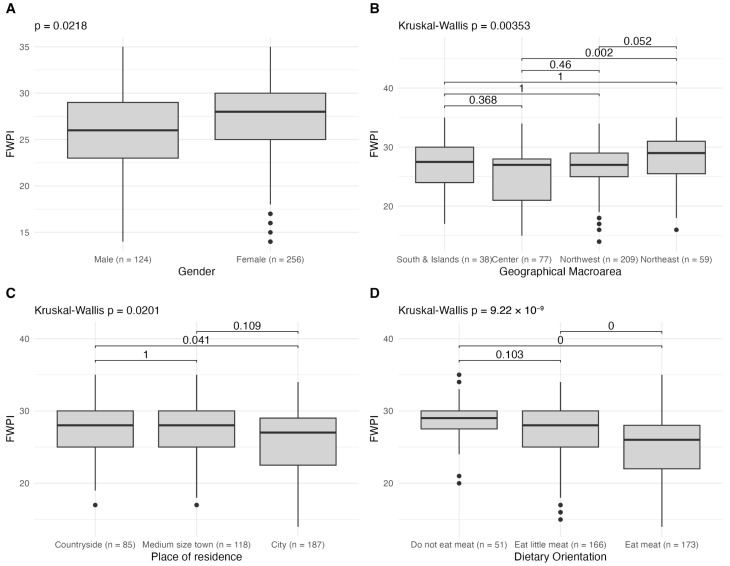
Boxplot of the Food Waste Prevention Index (FWPI) across different sociodemographic groups. (**A**) Comparison of FWPI scores by gender (male vs. female) using the Wilcoxon rank-sum test; (**B**) differences across geographical macro-areas (Northwest, Northeast, Center, South and Islands) with the Kruskal–Wallis test, followed by Dunn’s post hoc comparisons (Bonferroni-adjusted); (**C**) differences by place of residence (Countryside, Medium size town, City); and (**D**) differences by dietary orientation (do not eat meat, eat little meat, eat meat), both using Kruskal–Wallis with post hoc Dunn tests. Group sizes are indicated in parentheses on the *x*-axis. *p*-values from statistical tests are reported in each panel. Black points represent outliers.

The analysis of the differences in the FWPI across sociodemographic groups showed that females scored higher than men, indicating more frequent waste-reducing behaviors (*p* = 0.022) (Figure 1A). By macro-area, respondents living in the North-East exhibited higher FWPI values than those living in the Center (*p* < 0.001), with no statistically significant differences versus the other macro-areas (Figure 1B). A similar tendency was observed for place of residence: rural respondents showed higher scores than those living in cities (*p* = 0.02) (Figure 1C). Dietary orientation showed the clearest pattern: participants following meat-free or low-meat diets reported higher FWPI values than meat eaters (both *p* < 0.001) (Figure 1D). In contrast, we found no evidence that generation, education level, household composition, occupational status, or economic status were associated with the index.

### 3.3. Factors Predicting Food Waste Prevention Behaviors

A multiple linear regression tested predictors of the FWPI (Table 4). The model was statistically significant overall (F = 3.2, *p* < 0.00) and accounted for 15.4% of the variance in the index (R^2^ = 0.154; adjusted R^2^ = 0.106). Net of covariates, greater food waste knowledge was associated with more favorable behaviors (β = +0.57 SE = 0.22; *p* = 0.01). Compared with respondents who regularly eat meat, those who reported eating little meat (β = +1.6 SE = 0.47; *p* = 0.00) or not eating meat (β = +2.4 SE = 0.71; *p* = 0.00) scored higher. Household composition also mattered: people living alone (β = +1.93 SE = 0.85; *p* = 0.02) were associated with higher index scores (more virtuous behavior) relative to the respective reference category. Finally, residents of the North-East macro-area reported more sustainable behaviors than the other macro-areas (β = +1.71 SE = 0.73; *p* = 0.02).

### 3.4. Segmentation According to Different Food Waste Prevention Behaviors: Attitude and Group Characterization

Groups obtained from tertile values segmentation were, respectively, low_FWPI (≤25; n = 135), medium_FWPI (between 26 and 28; n = 108), and high_FWPI (≥28; n = 147). Groups did not significantly differ in sociodemographic composition regarding gender, generation, nationality, country of residence, educational level, family composition, and occupational and economic status (Table 1). Instead, the group composition indicated a significant difference regarding the Italian NUTS area [48], the context of living, and the diet. In particular, higher food waste prevention behavior was found in the North-East area, while virtuous behaviors were less present in the Central area. Moreover, food waste prevention behavior was more associated with rural contexts (countryside) and less with highly urbanized contexts (large cities). Finally, a more virtuous behavior was declared among flexitarians and vegans, while omnivores were more associated with a low FWPI.

Groups did not significantly differ in the frequency of use of social media to get informed about food (SocialMediaFoodInfoFreq), in the frequency of use of daily planner apps for food consumption (FoodPlannerAppFreq), or in the frequency of use of app dedicated to food delivery (FoodDeliveryAppFreq) (Table 1). Instead, the groups differed in the frequency of use of social media dedicated to information upon food waste (SocialMediaFoodWasteInfoFreq), in the frequency of use of apps dedicated to the grocery-planning (GroceryPlannerAppFreq), and in the frequency of use of apps such as TooGoodtoGo, Phenix, MyFood, and Wastemeter (FoodWasteAppFreq). In particular, a less virtuous behavior (lower FWPI) was associated with both a lower social media usage frequency (SocialMediaFoodWasteInfoFreq) and a lower use of the apps (FoodWasteAppFreq).

Considering the attitude of groups for food waste prevention and the sensitivity to information, some interesting differences across groups were found (Table 5). In particular, respondents with more virtuous behavior (higher FWPI) declared a higher awareness about vegetarian diets (Veg_informed, *p* = 0.009) and for principles related to circular economy (CircEcon_informed, *p* < 0.001), which may indicate a consistency between declared knowledge and actual behavior. Also, the consideration for short production chains was higher among respondents with a higher FWPI, indicating that awareness of food origin may significantly contribute to developing lower waste (FoodDistance_FoodCons, *p* = 0.007). Respondents with high FWPI declared higher attention to environmental sustainability in food choices, suggesting that environmentally aware consumers may engage in more sustainable consumption behaviors (EnvSust_FoodCons, *p* = 0.033). Respondents with high FWPI reported greater concern for environmental motivations behind vegetarian diets, suggesting that pro-environmental attitudes may correspond to an actual waste-reducing behavior (Environment_VegeDiet, *p* = 0.046). Similarly, they reported stronger agreement on animal welfare motivations for vegetarian diets, again highlighting a connection between ethical concern and practical behavior (AnimalWelfare_VegeDiet, *p* = 0.012). Respondents with high FWPI expressed the strongest willingness to reduce food waste, possibly reinforcing the awareness upon the importance of enhancing virtuous behaviors (reducing food waste) (WillingToReduceFW, *p* < 0.001). Overall, the findings suggest among consumers a consistency between the knowledge and awareness upon sustainability issues and actual behavior. Moreover, information sensitivity towards sustainability topics may effectively reduce food waste, highlighting the relevance of increasing sensitivity among consumers in order to develop effective interventions.

## 4. Discussion

### 4.1. Impact of Sociodemographic Factors on Digital Activism and Social Media Behavior Related to Food Waste

With reference to differences in food-related digital activism profiles, findings showed that sociodemographic factors significantly shaped digital activism and social media behaviors. Gender, generation, and economic conditions emerged as the strongest predictors of digital profiles, especially regarding food-related practices, while education and geography play a more limited role. Women were consistently more active across digital platforms, especially in seeking and sharing information on food and food waste. These data reflected a well-established finding in the literature: women show a higher propensity toward wellbeing, nutrition, ecological awareness, and the values of harmony, ethical universalism, care, and benevolence. This translates into more mindful and caring dietary styles [49,50,51] and into greater waste-conscious behaviors [52], also due to their stronger commitment to the management of household resources [44,53,54]. With regard to age factors, younger generations—particularly Generation Z—displayed the highest levels of digital engagement, especially on visually oriented platforms such as Instagram and TikTok, whereas older cohorts tended to adopt more selective and traditional patterns of use in agreement with previous studies [55,56,57,58]. Especially referring to food-related digital practices, despite Generation Z’s strong social media engagement and environmental awareness, the use of food waste reduction tools remained low. This reflected a generational pattern in which environmental consciousness loses priority when weighed against personal benefits [59]. Findings also showed that economic status differentiated the digital practices: less self-sufficient individuals—who largely represent the youngest segment of the surveyed population—tended to rely more on visual and entertainment platforms, whereas economically stable and fully self-sufficient users favored professional platforms such as LinkedIn, reflecting the link between economic stability and professional networking [60], as well as planning apps. Grocery-planning tools appeared polarized between economically non-self-sufficient benefit recipients, who showed the highest use likely for financial reasons, and self-sufficient individuals, who also reported relatively high use, probably reflecting greater awareness of food management and waste prevention—an attitude typically associated with higher economic independence [61]. Geographical patterns largely mirrored regional economic differences: LinkedIn showed the highest average use in the North-West and the lowest in the South, reflecting structural disparities in labor markets and the stronger presence of networking-oriented digital cultures in richer, northern regions. However, regional differences become negligible when considering social media uses related to food or food waste.

### 4.2. Impact of Sociodemographic Factors on Food Waste Prevention Behavior

From a psychometric perspective, the results of the Exploratory Factor Analysis provided important insights into the structure of food waste behaviors. The analysis revealed that items related to pre-store routines and meal planning (items 1–9) did not load coherently with items related to direct food management and disposal (items 10–16). Consequently, the final Food Waste Prevention Index (FWPI) was constructed using only the latter items, which specifically measure the actual execution of waste reduction (e.g., consuming leftovers). This distinction suggests that “preventative planning” and “direct waste behaviors” constitute two separate theoretical constructs. While planning is often considered a prerequisite for prevention, our data imply that high adherence to planning routines does not automatically align with the in-home management skills required to prevent waste at the consumption stage. By focusing the FWPI on items 10–16, this study isolates the outcome of waste prevention (not discarding food) rather than its antecedents (planning). Regarding the influence of external factors, our findings show systematic differences in the Food Waste Prevention Index by gender, area, and dietary style, while many classic sociodemographic traits were not associated.

Women’s FWPI higher scores (i.e., more waste-reducing behaviors) were consistent with studies reporting lower waste among women and stronger adherence to planning and reuse routines [54,62]. At the same time, category-specific analyses show that gender–waste links can vary (e.g., for fruit/vegetables in certain contexts), suggesting that gender gaps likely reflect different exposure to household food tasks rather than an inherent trait [44]. Spatial and contextual variables emerged as relevant: higher food waste prevention behavior was found in rural contexts and in the North-East region. This result highlighted the possible influence of local provisioning cultures, shopping infrastructures, and household consumption habits, where smaller communities and rural settings might foster more mindful consumption and reuse practices compared to large urban centers. This was in disagreement with a previous study indicating that rural households may exhibit larger purchasing units and less frequent shopping, leading to overstocking and eventual waste [63]; however, the territory considered by that study (Finland) may imply great differences compared to the Italian context. Instead, the higher food waste prevention behaviors in rural areas we found here plausibly gave evidence of higher alternative management channels outside cities (e.g., home composting, informal reuse) and higher time pressure in urban settings. In other terms, it seems less about “rurality” per se than about routines and opportunities: shopping networks (markets vs. hypermarkets), shopping frequency, storage/disposal space, and time constraints. Reviews also emphasize how infrastructure and domestic contexts (storage space, equipment, package sizes) shape practices and outcomes [17]. Studies in Italy and Lebanon, for example, documented strong contextual heterogeneity, where norms about meal preparation, frequency of eating out, and coping strategies (e.g., reuse of leftovers) shape household waste [40,45]. While in a study upon cross-country variability in household food waste behavior within the EU-27—including Italy—the intra-national differences across Italian regions were not explored [54]. More recent national studies suggested the existence of territorial differences in household food waste generation (e.g., [64,65]), although no consistent pattern has been established confirming lower/higher waste in the North-East of Italy. In this sense, the finding from our study indicating lower food waste in the North-East area represents an original contribution to the Italian context, pointing out potential regional disparities. Such differences might be associated with socio-economic characteristics, food availability, or consumption patterns specific to this area, as previously discussed in broader European analyses [54].

Dietary orientation showed the clearest association with food waste behaviors and practices. Respondents with higher virtuous behaviors (high_FWPI) were more often flexitarian or vegetarian, whereas lower virtuous consumers (low_FWI) were predominantly omnivorous. This aligns with prior research indicating that individuals adhering to vegetarian or flexitarian diets often hold stronger ethical and environmental values [66,67,68]. Therefore, the observed association between plant-based diets and higher Food Waste Prevention Index likely reflects a greater general environmental awareness and commitment to sustainability characteristic of these consumer segments, rather than being solely a function of diet composition per se. Regarding digital engagement, significant differences emerged for apps and social media related to food waste. The finding that Low_FWPI respondents reported lower use of social media and apps focused on food waste (e.g., TooGoodToGo, Phenix, MyFood, Wastemeter) and grocery-planning tools while High_FWPI tend to show the opposite was in line with some recent studies reporting that social media is an important factor influencing consumers’ attitude towards food waste behavior [69]. This result suggests that digital engagement, when directed toward sustainability-focused content, can support and possibly reinforce responsible consumption practices. However, given the correlational nature of the observed associations between digital activism and specific waste prevention practices or attitudes, any causal interpretation of the relationship between these two behavioral domains should be treated with caution.

Concerning attitude and sensitivity towards sustainable-related topics, the finding that High_FWPI individuals showed greater awareness of sustainability-related topics (e.g., reporting higher importance assigned to vegetarianism, circular economy, and local/short food chains) was in agreement with previous reports showing that environmentally conscious consumers report stronger engagement with sustainable diets [70]. High_FWPI individuals also displayed higher concern for environmental and animal welfare motivations behind vegetarian diets and a stronger willingness to reduce food waste. Conversely, low_FWPI respondents demonstrated lower sensitivity across these dimensions, indicating that limited awareness of sustainability issues may correspond to less virtuous consumption behaviors. This was recently reported also in the European Commission’s consumer segmentation study on food waste prevention conducted on over 25,000 citizens showing that a segment characterized by high self-reported food waste also showed a lack of awareness of ethical and environmental issues related to food waste [71].

The lack of associations of FWPI with generation, education, household composition, employment status, and economic status was consistent with diary studies and major reviews: when analysis shifts from “who we are” to “what we do” (planning, shopping, storing, cooking/reusing), explained variance increases and sociodemographic effects shrink or become inconsistent across countries and samples [39,42,72].

Overall, these results revealed a coherent pattern in which higher virtuous behavior toward food waste aligned with stronger environmental and ethical orientations, greater use of sustainability-oriented digital tools, and plant-based dietary preferences. Conversely, lower virtuous consumers appeared less engaged both attitudinally and behaviorally, suggesting that interventions should target awareness, digital education, and behavioral nudges to foster more consistent pro-sustainability practices.

### 4.3. Practical Implications and Limitations

The findings of this study offer actionable insights for policymakers and developers of digital interventions. First, the generational gap in digital activism suggests the need for segmented communication strategies: while Gen Z can be effectively engaged through short-video platforms (e.g., TikTok), older generations require more traditional digital channels (e.g., Facebook) or offline interventions focused on household management skills rather than activism. Second, the specific digital behavior of younger generations—characterized by high social media engagement but low use of food waste reduction tools—highlights a design gap. Since Gen Z tends to prioritize personal benefits and favors visual-entertainment platforms, current utility-focused apps fail to engage them. Consequently, developers should evolve from purely functional planning tools (which effectively serve economically stable users) to more gamified and visually engaging interfaces. To bridge the gap between Gen Z’s abstract awareness and action, apps must explicitly link waste reduction to tangible economic rewards and social gratification, transforming passive digital entertainment into active prevention habits. Finally, the strong link between waste prevention and reduced meat consumption suggests that anti-waste campaigns should be integrated with broader sustainable diet initiatives. Promoting plant-based habits could generate a positive spillover effect on waste reduction by leveraging shared environmental concerns.

The main limitation of the current study consisted of being based on self-reported responses. In fact, self-reported data are known to be affected by some bias such as social desirability and inaccuracies linked to self-esteem issues that interest epidemiological, clinical and consumer studies [73]. So, our data may underlie some degree of deviation from actual behavior, which, in most cases, leads surveys to underestimate food waste [28]. Finally, the operationalization of “digital activism” was based on the frequency of specific online behaviors (e.g., scrolling vs. reposting) rather than the qualitative depth of engagement. While we distinguished between passive consumption and active sharing, future research should explore the quality of interactions (e.g., critical commenting, community mobilization) to better capture the complexity of digital activism.

## 5. Conclusions

This study highlighted how sociodemographic factors and digital engagement can jointly influence household food waste prevention. Digital activism was strongly affected by gender, generation, and economic status, with smaller effects of education and geography. When considering self-reported behaviors, a higher food waste prevention behavior was associated with more sustainable dietary orientations (flexitarian and vegetarian) and higher engagement with digital tools dedicated to food waste prevention. Moreover, consumers with more virtuous behaviors declared greater awareness of sustainability principles (including circular economy, short food chains, and environmental motivations for vegetarian diets), together with a stronger willingness to reduce waste. These findings indicated an alignment between sustainability-related knowledge, ethical sensitivity, and actual waste-reducing practices, highlighting the importance of targeted communication strategies that enhance consumer awareness and information engagement. Overall, digital tools and appropriate information can be effective levers to enhance awareness, strengthen behavioral consistency, and promote responsible food management. Policies and interventions should integrate digital literacy, food education, and be tailored to support household food waste reduction.

## Figures and Tables

**Table 1:** Population and group characteristics for socio-demo variables and variables related to digital activism.

Type of Variable	Variable	Category	n	%	Low_ FWPI (n = 135)	Medium_FWPI (n = 108)		High_ FWPI (n = 147)			χ^2^	*p*-Value
Sociodemographic	*Gender*	Female	256	65.6	58.5		66.7		71.4		7.8	0.069
		Male	124	31.8	38.5		32.4		25.2			
		Non-binary/Other	10	2.6	3.0		0.9		3.4			
	*Generation (Age)*	Gen Z (18–29)	101	25.9	31.1		19.4		25.9		7.5	0.208
		Millennials (30–45)	159	40.8	36.3		50.9		37.4			
		Gen X (46–61)	114	29.2	28.9		25.9		32			
		Baby Boomers (62–78)	16	4.1	3.7		3.7		4.8			
	*Nationality*	Italian	380	97.4	97		98.1		97.3		0.1	0.625
		Other	10	2.6	3.0		1.9		2.7			
	*Living in Italy*	Permanently	373	95.6	95.6		95.4		95.9		1.2	0.417
		Occasionally	10	2.6	1.5		3.7		2.7			
		No	7	1.8	3.0		0.9		1.4			
	*Macro-area*	North-West	209	54.6	54.2	<	57	>	53.1	<	14.5	**<0.05**
		North-East	59	15.4	11.5	<	10.3	<	**22.8**	**>**		
		Center (C)	77	20.10	24.4	>	24.3	>	**13.1**	**<**		
		South and Islands	38	9.9	9.9	>	8.4	<	11	>		
	*Residence*	Countryside	85	21.8	21.0	<	15.7	<	**27.9**	**>**	10.0	**<0.05**
		Medium size town	118	30.3	29.0	<	27.8	<	33.3	>		
		City	187	47.9	50.0	>	**56.5**	**>**	**38.8**	**<**		
	*Educational level*	High	257	65.9	67.4		70.4		61.2		9.5	0.278
		Medium	120	30.8	2.2		4.6		3.4			
		Low	13	3.3	30.4		25		35.4			
	*Family members*	Alone	44	11.3	8.9		7.4		16.3		19.9	0.069
		Children	11	2.8	4.4		0.0		3.4			
		Friends/Flat mates	8	2.1	1.5		0.9		3.4			
		Parents or other family members	84	21.5	26.7		24.1		15.0			
		Partner and Children	119	30.5	30.4		34.3		27.9			
		Partner	113	29	26.7		30.6		29.9			
		Students/Coworkers	11	2.8	1.5		2.8		4.1			
	*Occupational status*	Employed	309	79.2	79.3		81.5		77.6		1.5	0.556
		Student	44	11.3	10.4		9.3		13.6			
		Unemployed/Not currently working	37	9.5	10.4		9.3		8.8			
	*Economic status*	I am fully self-sufficient/I support myself entirely	283	72.6	69.6		72.2		75.5		3.5	0.486
		I am partially self-sufficient/my family (relatives or partner) partially supports me	68	17.4	20.0		18.5		14.3			
		I am not self-sufficient/my family (relatives or partner) fully supports me	36	9.2	8.9		9.3		9.5			
		I am not self-sufficient/I receive public assistance	3	0.8	1.5		0.0		0.7			
Food habits	*Diet*	Eats meat (Omnivore)	173.0	44.5	59.3	**>**	50.0	>	26.5	**<**	37.2	**<0.05**
		Eats little meat (Flexitarian)	165.0	42.4	35.6	<	38.0	<	52.4	**>**		
		Does not eat meat (Vegetarian; vegan)	51.0	13.1	5.2	**<**	12.0	<	21.1	**>**		
Digital activism	*How frequently do you use social media to get informed about food?* (SocialMediaFoodInfoFreq)	Low	118	30.3	32.6		31.5		27.2		2.8	0.600
		Medium	141	36.2	38.5		35.2		34.7			
		High	131	33.6	28.9		33.3		38.1			
	*How often do you use social media to get information about food waste?* (SocialMediaFoodWasteInfoFreq)	Low	181	46.5	**54.8**	**>**	49.1	>	**37.0**	**<**	9.5	**<0.05**
		Medium	141	36.2	31.1	<	36.1	<	41.0	>		
		High	67	17.2	14.1	<	14.8	<	22.0	>		
	*How often do you use apps that offer you a “daily planner” for how much food to eat?* (FoodPlannerAppFreq)	Low	353.0	90.5	84.4		94.4		93.2		9.1	0.100
		Medium	21.0	5.4	8.9		2.8		4.1			
		High	16.0	4.1	6.7		2.8		2.7			
	*How often do you use apps that provide a “daily planner” for when you go grocery shopping?* (GroceryPlannerAppFreq)	Low	363.0	93.1	90.4	<	95.4	>	93.9	>	12.1	**<0.05**
		Medium	19.0	4.9	**8.9**	**>**	3.7	<	2.0	<		
		High	8.0	2.1	0.7	<	0.9	<	4.1	>		
	*How often do you use food delivery apps?* (FoodDeliveryAppFreq)	Low	307.0	78.7	72.6		77.8		85.0		6.6	0.200
		Medium	68.0	17.4	22.2		18.5		12.2			
		High	15.0	3.8	5.2		3.7		2.7			
	*How often do you use apps like TooGoodToGo, Phenix, MyFood, or Wastemeter?* (FoodWasteAppFreq)	Low	285.0	73.1	**83.0**	**>**	71.3	<	**65.3**	**<**	12.6	**<0.05**
		Medium	81.0	20.8	**14.8**	**<**	21.3	>	25.9	>		
		High	24.0	6.2	**2.2**	**<**	7.4	>	8.8	>		

The last two columns report the test of independence between the rows and the columns.

**Table 2:** Emerging digital activism (DA) profiles (% of respondents).

Used Platforms and Patterns of Use	Low DA (%)	Medium DA (%)	High DA (%)
Instagram	24.9	10.5	64.6
Facebook	47.2	22.3	30.5
X (Twitter)	88.5	6.2	5.4
Threads	90.3	5.9	3.8
LinkedIn	53.3	23.3	23.3
TikTok	81.3	8.5	10.3
Food-information activism (seeking info about food via social media)	30.3	36.2	33.6
Food-waste information activism	46.7	36.2	17.2
Daily meal-planner app use	90.5	5.4	4.1
Food delivery app use	78.7	17.4	3.8
Anti-waste app use (TooGoodToGo, Phenix, MyFood, Wastemeter)	73.1	20.8	6.2

**Table 3:** ANOVA results: Sociodemographic and digital activism practices.

			Mean	*p*-Value	Eta-Squared (η^2^)
*Gender*	Use of Instagram	Women	2.46	0.032	0.16
		Men	2.31		
		Non-binary/Other	1.90		
	Consuming contents (scrolling, watching, etc.)	Women	4.25	<0.001	0.52
		Men	3.56		
		Non-binary/Other	2.50		
	Reposting contents	Women	2.55	0.002	0.32
		Men	2.21		
		Non-binary/Other	1.80		
	Getting informed about food	Women	2.19	<0.001	1.12
		Men	2.15		
		Non-binary/Other	1.60		
	Getting informed about food waste	Women	1.80	0.001	0.35
		Men	1.51		
		Non-binary/Other	1.60		
	Use of apps to reduce food waste	Women	1.39	0.012	0.23
		Men	1.21		
		Non-binary/Other	1.20		
*Generation (Age)*	Use of Instagram	Gen Z (17–29)	3.15	<0.001	2.28
		Millennials (30–45)	2.54		
		Gen X (46–61)	2.34		
		Baby Boomers (62–78)	2.00		
	Use of Facebook	Gen Z (17–29)	1.51	<0.001	0.49
		Millennials (30–45)	2.33		
		Gen X (46–61)	2.38		
		Baby Boomers (62–78)	2.21		
	Use of TikTok	Gen Z (17–29)	1.60	<0.001	1.28
		Millennials (30–45)	1.22		
		Gen X (46–61)	1.14		
		Baby Boomers (62–78)	1.06		
	Checking one’s social media profile	Gen Z (17–29)	3.54	<0.001	1.45
		Millennials (30–45)	3.44		
		Gen X (46–61)	2.60		
		Baby Boomers (62–78)	2.34		
	Watching/Scrolling contents	Gen Z (17–29)	4.14	<0.001	1.52
		Millennials (30–45)	4.22		
		Gen X (46–61)	3.22		
		Baby Boomers (62–78)	4.09		
	Re-posting contents	Gen Z (17–29)	3.13	0.006	0.32
		Millennials (30–45)	2.32		
		Gen X (46–61)	2.32		
		Baby Boomers (62–78)	2.19		
	Getting informed about food	Gen Z (17–29)	2.24	<0.001	0.42
		Millennials (30–45)	2.08		
		Gen X (46–61)	2.23		
		Baby Boomers (62–78)	2.15		
*Education*	Use of LinkedIn	Low	1.00	<0.001	1.39
		Medium	1.39		
		High	2.28		
*Geographic Macro-area*	Use of Facebook	North-West	2.17	0.040	0.26
		North-East	2.21		
		Center	2.19		
		South	2.11		
		Islands	2.10		
	Use of X (former Twitter)	North-West	1.12	0.030	0.28
		North-East	1.14		
		Center	1.23		
		South	1.43		
		Islands	1.20		
	Use of LinkedIn	North-West	2.23	0.003	0.41
		North-East	2.09		
		Center	2.01		
		South	1.25		
		Islands	1.40		
*Economic Status*	Use of Instagram	Self-sufficient	2.32	0.009	0.03
		Partially self-sufficient	3.09		
		Not self-sufficient—supported by family	2.50		
		Receiving benefits	2.00		
	Use of LinkedIn	Self-sufficient	2.17	0.016	0.27
		Partially self-sufficient	1.60		
		Not self-sufficient—supported by family	1.39		
		Receiving benefits	1.00		
	Use of TikTok	Self-sufficient	1.21	<0.001	0.47
		Partially self-sufficient	1.46		
		Not self-sufficient—supported by family	2.01		
		Receiving benefits	1.00		
	Checking one’s social media profile	Self-sufficient	3.04	0.038	0.22
		Partially self-sufficient	3.50		
		Not self-sufficient—supported by family	3.50		
		Receiving benefits	2.33		
	Use of “meal planners” apps	Self-sufficient	1.16	0.008	0.03
		Partially self-sufficient	1.00		
		Not self-sufficient—supported by family	1.19		
		Receiving benefits	2.07		
	Use of “grocery shopping planners” apps	Self-sufficient	1.11	0.004	0.34
		Partially self-sufficient	1.01		
		Not self-sufficient—supported by family	1.03		
		Receiving benefits	1.67		

**Table 4:** Linear regression for predictors of the FWPI.

Term	Estimate	Std. Error	*p*-Value
(Intercept)	23.89	1.65	0.000
*FW_knowledge_Index*	**0.57**	**0.22**	**0.011**
*Gender [Female]*	−0.77	0.46	0.097
Male			
*Study Level [Low]*			
Medium	−0.74	1.24	0.553
High	−1.64	1.24	0.184
*Economic Status [I am fully financially self-sufficient; I support myself.]*			
I am partially self-sufficient/my family (relatives or partner) partially supports me	−0.56	0.70	0.425
I am not self-sufficient/my family (relatives or partner) fully supports me	−0.14	0.89	0.880
I am not self-sufficient/I receive public assistance	−4.91	4.21	0.244
*Diet [Eat meat]*			
Eat little meat	**1.60**	**0.47**	**0.001**
Do not eat meat	**2.39**	**0.71**	**0.001**
*Household members [Parents or other members of my family of origin.]*			
Children	−0.78	1.46	0.596
Partner	1.02	0.72	0.159
Friends/Flat mates	2.31	1.91	0.228
Students/Coworkers	2.40	1.45	0.099
Alone	**1.93**	**0.85**	**0.023**
Partner and Children	0.89	0.78	0.252
*Generation [Z]*			
Millennials	0.07	0.67	0.915
Gen X + Boomers	0.33	0.74	0.656
*Macro-area [Center]*			
North-East	1.71	0.73	0.021
North-West	0.76	0.56	0.176
South and Islands	1.38	0.83	0.098

Reference categories are in square brackets.

**Table 5:** Attitude of groups for food waste behavior and sensitivity to information.

Topic	Question	Category	Low_FWPI (n = 135)	Medium_FWPI (n = 108)	High_FWPI (n = 147)		*p*-Value
Sensitivity to information	How important do you think it is to be informed about the following topics? (Q4.1)	FW_informed	4.3 a	4.4 a	4.5 a	1.6	0.200
		**Veg_informed**	**3.3 ab**	**3.2 b**	**3.6 a**	**4.8**	**<0.05**
		**CircEcon_informed**	**4.1 b**	**4.5 a**	**4.4 a**	**7.2**	**<0.05**
		Seasonality_informed	4.3 a	4.6 a	4.5 a	2.5	0.100
Sensitivity to aspects of daily consumed food	How important do you consider the following aspects to the food you consume in a typical day? (Q4.3)	LocalProduct_FoodCons	3.8 a	4.0 a	4.1 a	2.4	0.100
		SeasonalProduct_FoodCons	4.2 a	4.3 a	4.4 a	2.4	0.100
		**FoodDistance_FoodCons**	**3.9 b**	**4.0 ab**	**4.2 a**	**5.0**	**<0.05**
		Organic_FoodCons	3.6 a	3.6 a	3.5 a	0.5	0.600
		Quality_FoodCons	4.3 a	4.3 a	4.2 a	0.6	0.600
		FairTrade_FoodCons	3.6 a	3.6 a	3.7 a	1.6	0.200
		**EnvSust_FoodCons**	**3.9 b**	**4.0 ab**	**4.2 a**	**3.4**	**<0.05**
		EconomicSust_FoodCons	3.7 a	3.8 a	3.9 a	1.6	0.200
		SocialSust_FoodCons	3.7 a	4.1 a	3.9 a	1.0	0.400
Importance given to drivers for vegetarian diet	How important would you evaluate the following reasons to adopt a more vegetarian diet?	Health_VegeDiet	4.3 a	4.4 a	4.3 a	0.1	0.900
		NewFlavors_VegeDiet	3.4 a	3.5 a	3.6 a	1.5	0.200
		WeightLoss_VegeDiet	2.9 a	2.9 a	2.7 a	0.8	0.400
		**Environment_VegeDiet**	**4.2 b**	**4.2 ab**	**4.4 a**	**3.1**	**<0.05**
		**AnimalWelfare_VegeDiet**	**3.7 b**	**3.9 ab**	**4.1 a**	**4.5**	**<0.05**
		Taste_VegeDiet	3.8 a	3.9 a	3.8 a	0.1	0.900
		Curiosity_VegeDiet	3.3 a	3.3 a	3.5 a	0.9	0.400
		LivingWithVege_VegeDiet	2.5 a	2.6 a	2.7 a	0.7	0.500
		Economic_VegeDiet	3.3 a	3.4 a	3.3 a	0.3	0.700
		ThirdWorld_VegeDiet	3.5 a	3.7 a	3.6 a	0.7	0.500
		Spiritual_VegeDiet	2.0 a	1.8 a	1.8 a	0.9	0.400
		SocialPressure_VegeDiet	2.3 a	2.2 a	2.2 a	0.4	0.700
		MeatTransition_VegeDiet	2.6 a	2.6 a	2.4 a	2.5	0.100
		None_VegeDiet	2.0 a	1.8 a	1.9 a	0.7	0.500
Willingness to reduce home food waste	How often are you willing to reconsider your consumption habits to reduce the food waste you produce at home?	**WillingToReduceFW**	**3.8 b**	**3.8 b**	**4.1 a**	**11.0**	**<0.05**

Lowercase letters indicate significant differences.

## Data Availability

The data presented in this study are available on request from the corresponding author due to privacy reasons.

## Supplementary Materials

The following supporting information can be downloaded at https://www.mdpi.com/article/10.3390/foods15030456/s1. File S1: Original version of the online survey.

## Abbreviations

The following abbreviations are used in this manuscript:

DA	Digital Activism
FWPI	Food Waste Prevention Index (FWPI)
FLW	Food Loss and Waste
FAO	Food and Agriculture Organization of the United Nations
SDG	Sustainable Development Goals
OECD	Organization for Economic Co-operation and Development
UNEP	The United Nations Environment Program
NUTS	Nomenclature of territorial units for statistics
