# Supplementary material for: Scrap the Food Waste: An Investigation of the Effect of Sociodemographic Factors and Digital Activism on Food Waste Prevention Behavior
Source: Foods. 2026 Jan 28;15(3):456. doi: 10.3390/foods15030456 (PMC12896848; doi:10.3390/foods15030456)
Supplement: Supplementary file 1 [file foods-15-00456-s001.zip › foods-4074151-supplementary.pdf]

Supplementary Materials

S.1 Original version of the online survey

Start of Block: Consenso

Q53 Benvenuta/o! Questo è un questionario realizzato da un team di ricercatori dell'Università di Scienze Gastronomiche di Pollenzo nell'ambito del progetto "Scrap the Food Waste". Lo scopo del questionario è raccogliere informazioni sullo spreco alimentare. La durata è di circa 10 minuti. Ti ringraziamo molto per il tuo interesse e la tua disponibilità! Puoi consultare l'informativa sul trattamento dei dati personali scaricandola a questo link: Informativa Per maggiori informazioni scrivere all'indirizzo mail l.torri@unisg.it Per maggiori informazioni sul progetto "Scrap the Food Waste" puoi consultare la pagina dedicata. Per sapere di più sull'Università di Scienze Gastronomiche consultare il sito ufficiale. Il progetto è finanziato dall'Unione europea. Le opinioni espresse appartengono, tuttavia, al solo o ai soli autori e non riflettono necessariamente le opinioni dell'Unione europea o dell’Agenzia esecutiva europea per europea per la salute e il digitale (HADEA). Né l'Unione europea né l'HADEA possono esserne ritenute responsabili.

- ○ Non Acconsento (4)
- ○ **Acconsento** (5)

Skip To: End of Survey If Q53 = Non Acconsento

End of Block: Consenso

Start of Block: Sociodemo

Q1.1 Seleziona il genere in cui ti identifichi

- ○ Donna (1)
- ○ Uomo (2)
- ○ Non-binario (3)
- ○ Preferisco non dirlo (4)

Q1.2 Età:

▼ (1) ... 76 (59)

Skip To: End of Survey If Q1.2 =
Skip To: End of Survey If Q1.2 = 76

Q1.3 Nazionalità:

- ◯ Italiana (1)
- ◯ Non Italiana (2)

Display this question:
If Q1.3 = Non Italiana

Q1.4 Nazionalità:

▼ Afghanistan (197) ... Zimbabwe (391)

Q1.5 Vivi in Italia?

- ◯ Sì, stabilmente (1)
- ◯ Sì, ma trascorro lunghi periodi (interi mesi) in un altro Paese (2)
- ◯ No, vivo stabilmente in un altro Paese (3)

Display this question:
If Q1.5 = Sì, stabilmente
Or Q1.5 = Sì, ma trascorro lunghi periodi (interi mesi) in un altro Paese

Q1.6 In quale regione d'Italia vivi?

▼ Abruzzo (1) ... Veneto (20)

Q1.7 La zona in cui vivi è:

- ○ Contesto Rurale/Campagna ((1)
- ○ Città medio-grande (10.000 - 70.000 abitanti)   (2)
- ○ Città (>70.000 abitanti) (3)

Q1.8 Titolo di studio conseguito

- ○ Licenza media o inferiore (1)
- ○ Diploma di scuola superiore (2)
- ○ Laurea triennale o post-diploma (3)
- ○ Laurea magistrale o master o dottorato (4)

Q1.9 Con chi vivi a casa?

- ○ Genitori o altri membri della famiglia di origine (1)
- ○ Figli (2)
- ○ Moglie/marito o partner convivente (3)
- ○ Moglie/marito o partner convivente e figli (7)
- ○ Amici/coinquilini (4)
- ○ Studenti o colleghi coinquilini (5)
- ○ Nessuno, vivo da solo/a (6)

Q1.10 Occupazione attuale:

- ◯ Occupato (full time, part-time, tempo determinato, occasionale, in proprio) (1)
- ◯ Studente (2)
- ◯ Non occupato/disoccupato (3)

Q1.11 Attuale condizione economica:

- ◯ Sono del tutto autosufficiente, mi mantengo da sola/o (1)
- ◯ Sono parzialmente autosufficiente, mi mantiene in parte la mia famiglia (parenti o partner) (2)
- ◯ Non sono autosufficiente, mi mantiene completamente la mia famiglia (parenti o partner) (3)
- ◯ Non sono autosufficiente, prendo un sussidio (4)

Q1.12 Abitudini alimentari:

- ◯ Onnivoro (mangio di tutto, sia prodotti animali che vegetali) (1)
- ◯ Flexitariano (mangio di tutto, ma cerco di ridurre la carne) (2)
- ◯ Vegetariano (non mangio la carne, ma mangio altri derivati animali, es. uova, formaggi, ecc.) (3)
- ◯ Vegano (non mangio alcun prodotto animale, né derivati) (4)

End of Block: Sociodemo

Start of Block: Attivismo Digitale

Q2.1 Con quale frequenza usi i seguenti social media?

	Mai (1)	Raramente (2)	A volte (3)	Spesso (4)	Sempre (5)
Instagram (1)	○	○	○	○	○
Facebook (2)	○	○	○	○	○
X (ex Twitter) (3)	○	○	○	○	○
Thread (4)	○	○	○	○	○
Linkedin (5)	○	○	○	○	○
TikTok (6)	○	○	○	○	○

Q2.2 Con quale frequenza controlli mediamente il tuo profilo sul tuo social media preferito?

- ○ Più di una volta all’ora (1)
- ○ Una volta all’ora (2)
- ○ Più di una volta al giorno (3)
- ○ Ogni giorno (4)
- ○ Una volta ogni 2 - 3 giorni (5)
- ○ Una volta alla settimana (6)

Q2.3 Con quale frequenza usi i social media per le seguenti attività?

	Mai (1)	Raramente (2)	A volte (3)	Spesso (4)	Sempre (5)
Guardare/leggere i post o i contenuti degli altri (1)	○	○	○	○	○
Creare post o contenuti (2)	○	○	○	○	○
Commentare post o contenuti creati da altri (3)	○	○	○	○	○
Ricondividere post o contenuti creati da altri (4)	○	○	○	○	○

Q2.4 Con quale frequenza posti contenuti sul tuo social media preferito?

○ Mai (1)

○ Ogni tanto (meno di una volta al mese) (2)

○ Qualche volta in un mese (3)

○ 1-2 volte a settimana (4)

○ Tutti i giorni (5)

Q2.5 Con quale frequenza usi i social media per informarti sul cibo?

○ Mai (1)

○ Raramente (2)

○ A volte (3)

○ Spesso (4)

○ Sempre (5)

Q2.6 Con quale frequenza usi i social media per informarti sullo spreco alimentare?

- Mai (1)
- Raramente (2)
- A volte (3)
- Spesso (4)
- Sempre (5)

Q2.7 Quanti follower/contatti hai sul tuo social media preferito?

- 0 - 500 (1)
- 501 - 1.000 (2)
- 1.001 - 5.000 (3)
- 5.001 - 10.000 (4)
- 10.001 - 25.000 (5)
- 25.001 - 100.000 (6)
- più di 100.000 (7)

Q2.8 Quanti account segui sul tuo social media preferito?

- 0 - 500 (1)
- 501 - 1.000 (2)
- 1.001 - 5.000 (3)
- 5.001 - 25.000 (4)
- 25.001 - 50.000 (5)
- 50.000 e più (6)

Q2.9 Con quale frequenza usi app che ti offrono un “daily planner” (programma quotidiano) di quanto cibo mangiare?

- Mai (1)
- Raramente (2)
- A volte (3)
- Spesso (4)
- Sempre (5)

Q2.10 Con quale frequenza usi app che forniscono un “daily planner” (programma quotidiano) per quando vai a fare la spesa?

- Mai (1)
- Raramente (2)
- A volte (3)
- Spesso (4)
- Sempre (5)

Q2.11 Con quale frequenza usi app di food delivery?

- Mai (1)
- Raramente (2)
- A volte (3)
- Spesso (4)
- Sempre (5)

Q2.12 Con quale frequenza usi app come TooGoodToGo, Phenix, MyFood, Wastemeter?

- Mai (1)
- Raramente (2)
- A volte (3)
- Spesso (4)
- Sempre (5)

End of Block: Attivismo Digitale

Start of Block: Conoscenza

Q3.1 Qual è la percentuale stimata della produzione alimentare globale sprecata ogni anno?

- 10% (0)
- 20% (0)
- 30% (1)
- 40% (0)

Q3.2 Quale settore contribuisce di più allo spreco alimentare globale?

- Settore agricolo e della logistica (0)
- Settore della ristorazione (0)
- Settore della vendita al dettaglio (0)
- Nuclei familiari (1)

Q3.3 Qual è lo spreco alimentare medio pro capite all’anno nei paesi ad alto reddito?

- Circa 10 kg (0)
- Circa 50 kg (0)
- Circa 90 kg (0)
- Circa 130 kg (1)

Q3.4 Quale delle seguenti azioni è più efficace per ridurre lo spreco alimentare domestico?

- Ignorare la data di scadenza e di consumo preferibile (0)
- Fare la lista della spesa, pianificando i pasti e la corretta conservazione dei prodotti (1)
- Acquistare in grandi quantità per cogliere l’opportunità delle offerte (0)
- Preparare porzioni più grandi e poi congelare tutto (0)

Q3.5 Qual è la principale causa dello spreco alimentare nel settore della vendita al dettaglio nei paesi ad alto reddito?

- Gestione degli ordini e difficoltà di prevedere le vendite con anticipo (0)
- Standard estetici sull'aspetto degli alimenti soprattutto se si tratta di prodotti freschi e freschissimi (frutta, verdura, formaggi, carni, ecc.) (1)
- Scaffali traboccanti e mancanza di infrastrutture di stoccaggio adeguate (0)
- Scarsa formazione professionale degli addetti alle vendite (0)

End of Block: Conoscenza

Start of Block: Sensibilità e Attitudine

Q4.1 Quanto ritieni importante essere informato/a sui seguenti temi?

	Per nulla importante (1)	Poco importante (2)	Moderatamente importante (3)	Abbastanza importante (4)	Molto importante (5)
Spreco alimentare (1)	○	○	○	○	○
Vegetarianismo (2)	○	○	○	○	○
Economia circolare e del recupero in cucina (3)	○	○	○	○	○
Stagionalità dei prodotti e provenienza (4)	○	○	○	○	○

Q4.2 Quanto ritieni importante il ruolo di questi ‘attori’ nella riduzione degli sprechi alimentari?

	Per nulla importante (1)	Poco importante (2)	Moderatamente importante (3)	Abbastanza importante (4)	Molto importante (5)
Distributori di alimenti (es. negozi, GDO, importatori) (1)	○	○	○	○	○
Produzione agricola (2)	○	○	○	○	○
Industria alimentare di trasformazione (3)	○	○	○	○	○
Terzo settore (es. ONG, social enterprises) (4)	○	○	○	○	○
Settore pubblico (es. città, stati) (5)	○	○	○	○	○
Me stessa/o (6)	○	○	○	○	○

Q4.3 Quanto ritieni importanti i seguenti aspetti per il cibo che consumi in un tuo giorno tipico?

	Per nulla importante (1)	Poco importante (2)	Moderatamente importante (3)	Abbastanza importante (4)	Molto importante (5)
È un prodotto locale/regionale (1)	○	○	○	○	○
È un prodotto stagionale (2)	○	○	○	○	○
Proviene da vicino (breve distanza di trasporto) (3)	○	○	○	○	○
È un prodotto biologico (4)	○	○	○	○	○
È un prodotto di qualità (5)	○	○	○	○	○
È un prodotto equo e solidale (6)	○	○	○	○	○
È un prodotto attento alla sostenibilità ambientale (7)	○	○	○	○	○
È un prodotto attento alla sostenibilità economica (8)	○	○	○	○	○
È un prodotto attento alla sostenibilità sociale (9)	○	○	○	○	○

Q4.4 Quanto ritieni importanti le seguenti ragioni se volessi fare una dieta più vegetariana?

	Per nulla importante (1)	Poco importante (2)	Moderatamente importante (3)	Abbastanza importante (4)	Molto importante (5)
La mia salute (1)	○	○	○	○	○
La volontà di scoprire nuovi sapori (2)	○	○	○	○	○
La necessità di ridurre il mio peso (3)	○	○	○	○	○
L'ambiente/il clima (4)	○	○	○	○	○
Il benessere animale (5)	○	○	○	○	○
È buono (6)	○	○	○	○	○
La mia curiosità (7)	○	○	○	○	○
Persone vegetariane con cui vivo (8)	○	○	○	○	○
È economico (9)	○	○	○	○	○
Il terzo mondo (10)	○	○	○	○	○
Credenze spirituali/religiose (11)	○	○	○	○	○
Pressione sociale (12)	○	○	○	○	○
Mangiare carne è una fase transitoria (13)	○	○	○	○	○
Nessuna ragione (14)	○	○	○	○	○

Q4.5 Quanto spesso sei disposto/a a riconsiderare le tue abitudini di consumo per ridurre lo spreco alimentare che produci in casa?

- Mai (1)
- Raramente (2)
- A volte (3)
- Spesso (4)
- Sempre (5)

End of Block: Sensibilità e Attitudine

Start of Block: Comportamenti e Pratiche

Q5.1 Rispetto ai seguenti temi, quanto ti capita di condividere informazioni, opinioni, punti di vista, su dispositivi digitali e social network?

	Mai (1)	Raramente (2)	A volte (3)	Spesso (4)	Sempre (5)
Spreco alimentare (1)	o	o	o	o	o
Vegetarianismo (2)	o	o	o	o	o
Economia circolare e del recupero in cucina (3)	o	o	o	o	o
Stagionalità dei prodotti e provenienza (4)	o	o	o	o	o

Q5.2 Rispetto ai seguenti temi, quanto ti capita di leggere notizie, approfondimenti, etc., su dispositivi digitali e social network?

	Mai (1)	Raramente (2)	A volte (3)	Spesso (4)	Sempre (5)
Spreco alimentare (1)	○	○	○	○	○
Vegetarianismo (2)	○	○	○	○	○
Economia circolare e del recupero in cucina (3)	○	○	○	○	○
Stagionalità dei prodotti e provenienza (4)	○	○	○	○	○

Q5.3 Pianifichi i pasti prima di fare la spesa?

○ Mai (1)

○ Raramente (2)

○ A volte (3)

○ Spesso (4)

○ Sempre (5)

Q5.4 Crei e segui una lista della spesa?

○ Mai (1)

○ Raramente (2)

○ A volte (3)

○ Spesso (4)

○ Sempre (5)

Q5.5 Con quale frequenza controlli il frigorifero e la dispensa per verificare la presenza di prodotti prossimi alla scadenza e da utilizzare a breve?

- ◯ Mai (1)
- ◯ Raramente (2)
- ◯ A volte (3)
- ◯ Spesso (4)
- ◯ Sempre (5)

Q5.6 Quanto spesso conservi gli avanzi in frigorifero o freezer per utilizzarli in un secondo momento?

- ◯ Mai (1)
- ◯ Raramente (2)
- ◯ A volte (3)
- ◯ Spesso (4)
- ◯ Sempre (5)

Q5.7 Quando cucini, quanto spesso prepari solo la quantità di cibo che ipoteticamente verrà consumata, invece di prepararne in più per un pasto programmato in futuro (ad esempio, il pranzo o la cena del giorno successivo)?

- ◯ Mai (1)
- ◯ Raramente (2)
- ◯ A volte (3)
- ◯ Spesso (4)
- ◯ Sempre (5)

Q5.8 Quanto spesso controlli la data di scadenza (entro e non oltre) o il termine minimo di conservazione (preferibilmente entro) prima di acquistare gli alimenti?

- ○ Mai (1)
- ○ Raramente (2)
- ○ A volte (3)
- ○ Spesso (4)
- ○ Sempre (5)

Q5.9 Prepari ogni giorno piatti diversi?

- ○ Mai (1)
- ○ Raramente (2)
- ○ A volte (3)
- ○ Spesso (4)
- ○ Sempre (5)

Q5.10 Con quale frequenza vai a fare la spesa?

- ○ Mai (1)
- ○ Raramente (2)
- ○ A volte (3)
- ○ Spesso (4)
- ○ Sempre (5)

Q5.11 Quanto spesso mangi fuori casa?

- Mai (1)
- Raramente (2)
- A volte (3)
- Spesso (4)
- Sempre (5)

Q5.12 Getti via gli avanzi perché nessuno vuole mangiarli?

- Mai (1)
- Raramente (2)
- A volte (3)
- Spesso (4)
- Sempre (5)

Q5.13 Quanto spesso trovi difficile preparare un pasto con ingredienti avanzati o con il cibo che hai a portata di mano?

- Mai (1)
- Raramente (2)
- A volte (3)
- Spesso (4)
- Sempre (5)

Q5.14 Quanto spesso ti senti incerto sulla sicurezza del consumo di alimenti vicini alla data di scadenza?

- ◯ Mai (1)
- ◯ Raramente (2)
- ◯ A volte (3)
- ◯ Spesso (4)
- ◯ Sempre (5)

Q5.15 Acquisti cibo solo perché è in offerta o perché sembra invitante, anche se non è necessario?

- ◯ Mai (1)
- ◯ Raramente (2)
- ◯ A volte (3)
- ◯ Spesso (4)
- ◯ Sempre (5)

Q5.16 Dimentichi il cibo in frigorifero o in freezer fino a che non diventa troppo vecchio per essere consumato?

- ◯ Mai (1)
- ◯ Raramente (2)
- ◯ A volte (3)
- ◯ Spesso (4)
- ◯ Sempre (5)

Q5.17 Quanto è probabile che scarti gli alimenti solo in base all'aspetto (ad esempio, lievi ammaccature su frutta e verdura)?

- Mai (1)
- Raramente (2)
- A volte (3)
- Spesso (4)
- Sempre (5)

Q5.18 Getti via gli alimenti confezionati che hanno superato la data di scadenza, anche se non aperti o senza averli provati?

- Mai (1)
- Raramente (2)
- A volte (3)
- Spesso (4)
- Sempre (5)

End of Block: Comportamenti e Pratiche
